# Hijacking intercellular trafficking for the spread of protein aggregates in neurodegenerative diseases: a focus on tunneling nanotubes (TNTs)

**DOI:** 10.20517/evcna.2023.05

**Published:** 2023-03-09

**Authors:** Ranabir Chakraborty, Sevan Belian, Chiara Zurzolo

**Affiliations:** ^1^Institut Pasteur, Université Paris Cité, CNRS UMR 3691, Membrane Traffic and Pathogenesis, Paris F-75015, France.; ^2^Université Paris Saclay, Gif-sur-Yvette, Paris 91190, France.; ^3^Department of Molecular Medicine and Medical Biotechnology, University of Naples Federico II, Naples, Italy.; ^#^Authors contributed equally.

**Keywords:** Tunneling nanotubes, intercellular communication, neurodegenerative diseases

## Abstract

Over the years, the influence of secretory mechanisms on intercellular communication has been extensively studied. In the central nervous system (CNS), both trans-synaptic (neurotransmitter-based) and long-distance (extracellular vesicles-based) communications regulate activities and homeostasis. In less than a couple of decades, however, there has been a major paradigm shift in our understanding of intercellular communication. Increasing evidence suggests that besides secretory mechanisms (via extracellular vesicles), several cells are capable of establishing long-distance communication routes referred to as Tunneling Nanotubes (TNTs). TNTs are membranous bridges classically supported by F-Actin filaments, allowing for the exchange of different types of intracellular components between the connected cells, ranging from ions and organelles to pathogens and toxic protein aggregates. The roles of TNTs in pathological spreading of several neurodegenerative conditions such as Prion diseases, Alzheimer’s disease (AD), Parkinson’s disease (PD), and Huntington’s disease (HD) have been well established. However, the fragile nature of these structures and lack of specific biomarkers raised some skepticism regarding their existence. In this review, we will first place TNTs within the spectrum of intercellular communication mechanisms before discussing their known and hypothesized biological relevance *in vitro* and *in vivo *in physiological and neurodegenerative contexts. Finally, we discuss the challenges and promising prospects in the field of TNT studies.

## INTRODUCTION

### Mechanisms of intercellular communication: extracellular vesicle vs. membrane protrusions

**Figure 1:**
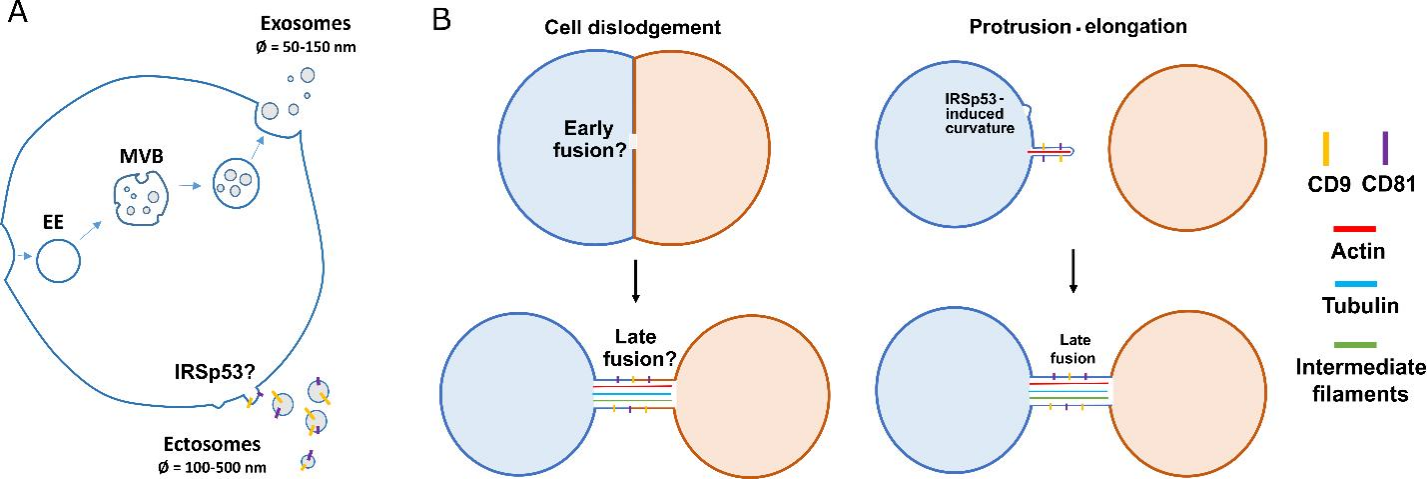
Formation mechanisms of EVs and TNTs. (A) EVs are generated via different pathways. Generation of exosomes require inner budding within EE maturing into MVBs, whereas ectosomes form via negative membrane curvature-induced budding at the plasma membrane. Both the tetraspanins CD9 and CD81 are components of ectosomal membranes and are found on TNTs. (B) TNTs can be formed via different mechanisms, viz. cell dislodgement (left panels), wherein cells that come in contact with each other leave behind a tubular connection when they move apart, and protrusion-elongation (right panels), where one cell, following negative membrane curvature, actively extends a protrusion towards a neighbouring cell to eventually form a functional connection.

In animals, intercellular communication can occur at different scales. Some mechanisms rely on the release of secretory molecules in the extracellular environment upon the fusion of intracellular vesicles with the plasma membrane. In the peculiar case of hormones, these molecules can travel through the circulatory system and reach membrane receptors of distant cells. However, the signal spreading often takes place within a few hundred microns only, as it relies on the local concentrations of the signaling molecules, which decrease in an exponential fashion as diffusion in tissues occurs^[1]^. Other mechanisms involve the transport of extracellular vesicles (EVs). Encapsulated ions, proteins, RNAs, or even the lipid and protein content of the vesicles itself can trigger intracellular responses and phenotypic changes following their uptake by neighbor cells. Such responses usually include regulation of pro-/anti-inflammatory pathways, as described in excellent reviews^[2,3]^. Consequently, this mode of communication involves material transfer between cells and can therefore be used by pathogens as a route for spreading, as has been demonstrated for the Hepatitis C Virus (HCV), or for the aggregate-prone prion protein Scrapie (PrP^Sc^)^[4-6]^ and for different protein aggregates accumulating in neurodegenerative diseases (NDs), as will be discussed subsequently. Additionally, EVs represent important regulators of the immune response by transporting antigens to immune cells^[7]^. Through different formation mechanisms, the composition of these vesicles is finely tuned to have them interact with surrounding cells in a specific or non-specific manner to induce a wide range of responses^[8-10]^. Despite the development of new promising tools enabling single-vesicle analysis^[11]^, we are only beginning to understand the diversity of EVs. In fact, the current limitations of the methods used to purify a single population led the International Society for Extracellular vesicles to recommend the use of the terminologies “small EVs” (< 200 nm) and “large EVs” (diameter > 200 nm)^[12]^. Furthermore, different formation mechanisms for EVs have been identified. Exosomes (diameter < 50-150 nm), for example, form within the lumen of early endosomes (EE), the latter eventually maturing into multivesicular bodies (MVBs). Upon fusion of MVBs with the plasma membrane, exosomes are released in the extracellular environment. On the other hand, ectosomes (also called microvesicles with a diameter of 100-500 nm) form via direct budding from the plasma membrane [Figure 1A]. Importantly, for both exosomes and ectosomes, luminal and membrane composition greatly varies within a single cell and across different cell types, possibly conferring different functions on them^[13]^. Another subtype of EVs, called migrasomes, has been described to have putative roles in intercellular communication. Migrating cells leave behind a trail of these organelles containing Tetraspanin-enriched microdomains^[14]^, which can then be taken up by other cells, thereby transferring their contents, a phenomenon referred to as migracytosis^[15]^.

In addition to secretory vesicles, other intercellular communication mechanisms effective at smaller ranges rely on direct cell-to-cell contact. Filopodia, for example, are very dynamic short cellular protrusions (usually less than 5 µm in length) that allow intercellular signaling without material exchange. Such signaling is mediated by surface receptors present on the filopodial tip, such as Cadherins^[16]^ or Integrins^[17]^. The binding of such receptors directly at the tip or indirectly (through the transduction of mechanical force(s) via the Actin bundle to the base of the filopodial shaft) triggers Ras superfamily-mediated downstream signaling, subsequently leading to various cellular responses^[17,18]^. Thus, by regulating expression, subcellular localization and activation of these receptors or ligands, cells have a way to sense and communicate with their microenvironment. Filopodia also favor cell migration, playing major roles in cancer and wound healing^[19,20]^. Some specialized filopodia-like protrusions are cytonemes, which can reach up to 700 µm long, allowing the transfer of signaling molecules between cells at contact sites called morphological synapses^[21-23]^. These close-ended specialized structures were shown to play key roles in development as they allow the spreading of morphogens, for example, in *Drosophila melanogaster* during air sac primordium’s development, through the transport of Decapentaplegic (DPP) or Fibroblast Growth Factor (FGF) receptors^[24]^. Importantly, cytonemes have been observed in different *in vivo* models, from worms to mammals^[22,25,26]^.

The latest addition to the family of membrane protrusions allowing intercellular communication is represented by Tunneling nanotubes (TNTs), which are F-Actin-positive, open-ended bridges that connect the cytoplasm of cells up to a hundred micrometers apart, with diameters varying between 50 nm-900 nm. In 2D culture, they are observed as hovering over the substrate. Most importantly, TNTs allow the transfer of various cargoes between cells, such as ions, proteins, RNAs, as well as organelles^[27-30]^. TNTs can also be close-ended (containing gap junctions) structures that allow electrical coupling between cells through Ca^2+^ signaling both *in vivo* and *in vitro*^[31-34]^. These structures have been identified in various cell types *in vitro*, such as epithelial^[35]^, immune^[36,37]^, cardiac^[38]^ and neuronal cells^[30]^, allowing both homotypic and heterotypic connections. More recently TNT-like structures were found in various *in vivo* animal models^[32,34]^. Depending on the cell type, microtubules and, to a lesser extent, intermediate filaments have also been reported inside TNTs, usually associated with an increase in diameter^[39]^. These structural variations have led to the annotations of “thin” (only F-Actin) and “thick” (F-Actin and microtubule/intermediate filaments) TNTs, which are thought to be associated with functional differences, likely related to the prevalent role these filaments can play in material transfer^[40]^.

Classical open-ended TNTs differ fundamentally from other intercellular communication mechanisms as they allow cytoplasmic continuity between two cells^[41,30]^. By connecting the cytosol of the cells, they allow transfer of material through both active transport and passive diffusion. The formation of TNTs is upregulated in stressful conditions such as hypoxia or serum deprivation and was shown to be promoted by the NF-*κ*B pathway^[32,42-44]^. Overall, these observations strongly suggest an implication of TNTs in the control of inflammation in physiological and pathological processes *in vivo*. Similar to EVs, TNTs appear essential in maintaining homeostasis through communication and cooperation between cells through material exchange. However, the other side of that coin is the hijacking of these structures by pathogens, favoring the spreading of diseases. Viruses such as HIV-1^[45]^, SARS-CoV-2^[46]^ or Herpes^[47]^ were shown to exploit TNTs as a way to propagate while evading the immune system. TNTs were also proposed to be involved in the spreading of various neurodegenerative pathologies because they are able to mediate the propagation of aggregate-prone proteins accumulating in different NDs (reviewed by Soraya Victoria and Zurzolo^[48]^). Finally, cancer cells also appear to use TNTs as a way to survive chemo- and radio-therapy and adapt to their microenvironment^[49-52]^. In this review, we summarize the structural and functional characteristics of TNTs and their role in the propagation of aggregate-prone proteins in different NDs.

## MECHANISM OF TNT FORMATION

### Molecular players of cytoskeletal regulation in TNT formation

Two main mechanisms of TNT formation have been proposed: cell dislodgement and protrusion-elongation [Figure 1B]. So far, these formation mechanisms have not been correlated to any difference in structure or functionality. As mature neurons are post-mitotic in nature and exhibit low migratory phenotype past embryonic development, cell dislodgement does not appear to be a favored formation mechanism for TNTs between neuronal cells, which is consistent with previous observations in catecholaminergic-derived neuronal cell line (CAD cells)^[53,54]^.

Therefore, hereafter we focus on the protrusion-elongation mechanism.

Other Actin-based protrusions have been described and studied before the discovery of TNTs, such as filopodia, microvilli, or stereocilia. Interestingly, common players have been identified in the formation mechanism of these structures, hinting us towards the probable actors involved in TNT formation. Supported by current literature^[53]^, one of the models describing the critical steps involved in TNT formation through protrusion-elongation begins with a signaling cascade leading to the activation of Rho GTPases. This in turn leads to the activation and clustering of membrane-bending proteins to locally induce negative membrane curvature, which is associated with the recruitment of Actin polymerizers and Actin bundlers to create and elongate a bundle of Actin filament that will push the membrane and grow the protrusion^[55]^. In the past years, some actors shown to positively regulate TNT formation have strengthened this model, such as the G-protein Rab35^[56]^, the I-BAR protein IRSp53^[53]^, or the Actin bundler Eps8^[57]^. Eventually, fusion occurs at the tip of the TNT, probably through a process of activation and recruitment of adhesive proteins associated with Actin polymerization to drive the force required to break the membrane tension, as observed in myoblast fusion^[58]^. Consistent with this hypothesis, very recent data support the role of the N-Cadherin-*α*-Catenin complex, as well as of tetraspanins (CD9 and CD81) in the process of fusion with the receiving cell [Figure 1B]^[30,59,60]^. Following the formation of the structure, motor proteins would mediate the transport of cargoes^[54,61]^. The nature of cargoes transferred from a donor to an acceptor cell seems to be a well-organized event, with the involvement of molecular motors, intracellular/extracellular components, type and health of the connected cells, and potentially several other influential factors that are yet to be discovered. The functional nature of TNTs will be discussed subsequently.

What regulates the formation of a TNT with an acceptor cell and whether this process is random or guided remains largely unknown. In rat hippocampal neurons and astrocytes, p53 leads to caspase-3 activation, subsequently leading to the cleavage of the calcium-binding protein S100A4 in TNT-initiating cells. This consequently results in an extracellular gradient of S100A4 which was shown to direct TNT formation towards other cell^[62]^. It is yet the only known mechanism of guidance of TNTs via chemotactic cues, but it leads us to think that the general directionality of TNT growth might be regulated through similar processes.

### Mechanism of filopodia versus TNT formation

From a structural point of view, TNTs present striking homologies with filopodia^[55]^. Consistent with that observation, many of the molecular actors that have been identified so far to promote TNT formation also play central roles in filopodial formation, such as Rab35, the unconventional Myosin X, Eps8 as well as IRSp53^[53,54,56]^. Therefore, a central question of the field lies in understanding whether TNTs originate from the differentiation of a subset of filopodia as it is believed to be the case for cytonemes^[63]^, or if they are distinct structures from the beginning. Recent evidence obtained using cryo-correlative light and electron microscopy (cryo-CLEM) of TNTs and VASP-induced filopodia in murine neuronal-like (CAD) cells leads us to hypothesize that TNTs are unique structures from the beginning of their formation^[30]^.

Cryo-CLEM showed that the Actin bundle within filopodia and TNTs arrange in hexagonal arrays with a comparable average distance separating the filaments (~4.7 nm for filopodia, ~5.5 nm for TNTs). This suggests redundancy in the Actin bundlers present in both structures. However, filopodia in CAD cells are individual close-ended protrusions, while TNTs imaged at high resolution consist of a bundle of small open-ended tubes called iTNTs (individual TNTs) running parallel to each other^[30]^. Additionally, TNTs *in vitro* are always non-adherent to the substrate, suggesting a different protein and lipid composition of the membrane of the protrusions, or a different activation pattern of adherent proteins. These differences *per se* are not sufficient to allow us to exclude the possibility that TNTs differentiate from preformed filopodia. However, Actin filaments within each iTNT in the bundle run uninterrupted all along the imaged areas (1.2-1.5 µm), and F-Actin continuity within filopodia is interrupted every 0.3 to 1.1 μm. These results, associated with the fact that TNTs reach far greater distances compared to filopodia, suggest that different Actin polymerizers with different processivities are responsible for the growth of protrusions. If so, it would mean TNTs and filopodia arise from different molecular actors early during their formation^[57]^.

### Mechanism of ectosomes versus TNT formation

Surprisingly, TNTs also share interesting similarities with ectosomes. During early formation, they both rely on the formation of microdomains at the plasma membrane regulating the recruitment of protein complexes leading to negative membrane curvature. In fact, knock-down of IRSp53 was shown to decrease ectosome shedding^[64,65]^. As such, the involvement of IRSp53 in TNT formation^[53]^ suggests the potential convergence of signaling pathways in regulating ectosomes and TNTs [Figure 1]. Additionally, they have in common the presence of tetraspanins such as CD9 and CD81, known to interact with integrins and to play a major role in sperm-egg fusion during fertilization^[66]^ [Figure 1]. As both ectosomes and TNTs share a similar fate (fusing with a neighbor cell), these membrane proteins could be involved in the same process. Recent investigation on the proteome of TNTs versus EVs has shown the presence of specific but also common components^[60]^. Thus, it is possible that from an evolutionary point of view, TNTs emerge from molecular pathways involved in filopodial formation and ectosome shedding. Further investigation will be necessary to test this intriguing hypothesis.

## FUNCTIONAL ROLES OF TUNNELING NANOTUBES

**Figure 2:**
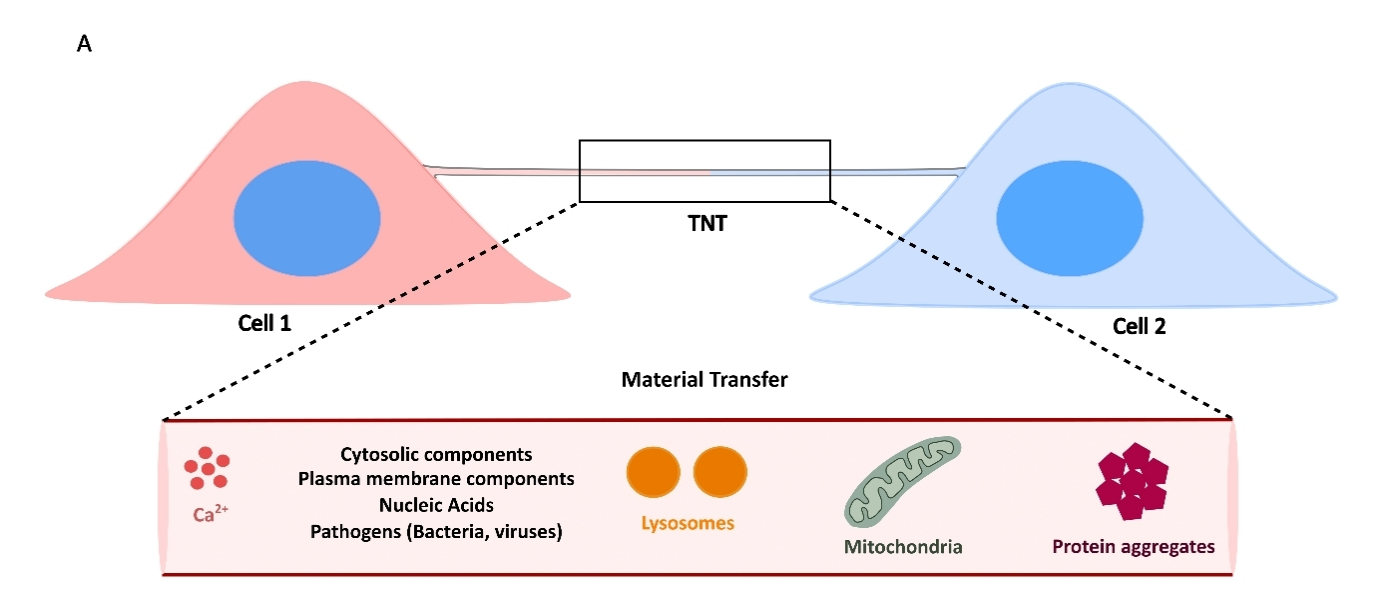
Functional nature of TNTs is defined by their ability to facilitate material transfer between connected cells. A: Cells connected by TNTs can allow for exchange of various intracellular materials, such as ions, cytosolic and plasma membrane components, nucleic acids such as messenger and/or regulatory RNAs, organelles such as lysosomes and mitochondria, and cytotoxic protein aggregates.

The major characteristic point that distinguishes TNTs from any other kind of cellular protrusions is their ability to transfer cargoes between connected cells. The different cytosolic and/or engulfed materials that have been reported to be transferred (ions, vesicles, nucleic acids, organelles, pathogens, proteins and proteinaceous aggregates) suggest critical roles of TNTs in maintenance of homeostasis within the cellular network, as well as in spreading of pathologies^[28,59,67,68] ^[Figure 2]. Movement across a nanotube can occur either uni-directionally or bi-directionally, depending on the context. As a plausible mechanism for diluting the effects of stress, an unhealthy cell can transfer materials such as damaged organelles or protein aggregates to the connected cell in a unidirectional manner^[69]^. However, this unidirectional transfer can also lead to spread of neurodegenerative pathologies such as PD and AD, wherein movement of *α*-Synuclein (*α*-Syn) and Tau aggregates to an acceptor (or host) cell can initiate seeding of new aggregates^[70,71]^. On the other hand, a healthy cell can transfer unidirectionally functional components to the damaged cell as a mechanism of alleviating stress^[72]^. Bidirectional transfer aims towards mutual exchange of materials and has been shown to occur between different cell types^[73,74]^.

### Transfer of ions and electrical coupling via TNTs 

The first demonstration of TNTs providing a route for transfer of Ca^2+^ between connected cells was between THP-1 monocytes and dendritic cells, eventually generating an immune response in dendritic cells, mimicking what can be observed upon response to bacteria^[75]^. Eventually, Ca^2+^ transfer via TNTs was also observed between RAW264.7 macrophages^[76]^. In what can be considered as a significant advancement towards understanding the basis of such transfer, Smith *et al*. observed the involvement of inositol triphosphate (IP_3_) receptors along the length of TNTs connecting SH-SY5Y and HEK cells. This provides evidence for propagation of Ca^2+^ as an active process mediated by successive phases of Ca^2+^ release followed by Ca^2+^-induced Ca^2+^ release (CICR), and not mere passive diffusion of ions^[77]^. Besides open-ended TNTs, Ca^2+^ can travel between cells via close-ended TNTs as well, with gap junctions allowing for their entry to the other cell, as has been demonstrated by the presence of Connexin43 (Cx43) at one end of the TNTs^[78]^. Ca^2+^-mediated electrical coupling via TNTs and concomitant expression of neuronal Cx43 have also been observed between neurons and astrocytes at an early stage (5 hours in co-culture) but not after 24 hours^[31]^. As such, the involvement of TNTs in development and migration of neurons, even before the establishment of synapses, might be of critical importance^[29]^. However, TNTs between Jurkat T-cells have been shown to be incompetent in propagating Ca^2+^ between connected cells, suggestive of differential functional properties of TNTs in a cell type-dependent manner^[79]^. Besides *in vitro* conditions, TNTs between pericytes of murine retina (IP-TNTs) connect nearby capillaries and coordinate neurovascular coupling, a phenomenon that is lost upon ischemia-induced IP-TNT damage^[34]^. This opens new directions of studies into neuron-glia interactions and electrical coupling via TNTs in healthy and disease conditions, with special emphasis on neurodevelopmental disorders and NDs.

### Transfer of signals and nucleic acids

In addition to Ca^2+^ mediated signaling, TNTs are capable of transferring several components of different signaling pathways. Initial discoveries of such transfer were made in immune cells, where it was observed that activation of Fas receptors on T-cells promoted TNT formation with neighboring T-cells, leading to movement of membrane-bound FasL and active caspase-3^[80]^. Natural killer (NK) cells can form functional nanotubes upon their activation and eventually cause cytotoxicity of target cells. Accumulation of DAP10, and Major Histocompatibility Complex (MHC) class-I chain-related protein A (MICA) at the tip of nanotubes between NK cells and target cells was sufficient for such immune interaction^[81]^. The cytoplasmic stain Calcein-AM was also observed to be transferred between mesenchymal multipotent stromal cells (MMSCs) and rat renal tubular cells (RTCs) in a co-culture system^[73]^. Similarly, other cytoplasmic stains and dyes like CFSE and Lucifer Yellow have also been shown to move between cells via TNTs^[75,80]^. Notably, cytosolic EGFP has recently been reported to move from layer I-III cortical astrocytes to layer V neurons via tunneling nanotubes^[82]^.

Finally, movement of nucleic acids between cells via TNTs provides a mechanism of regulation of gene expression at a global level of connected cells. Several forms of nucleic acids have been reported to reach target cells via the route of nanotubes, such as mitochondrial DNA^[72,83]^, messenger RNA^[84,85]^, viral RNAs^[35,46,86]^, and regulatory miRNAs^[87-89]^. This potentially allows for the donor cell to regulate the transcriptomic and metabolomic profiles of the target cells, with critical implications in neurological pathologies, and other conditions such as cancers, involving regulatory nucleic acids.

### Plasma membrane component, intracellular vesicles and organelles

Besides exchanging cytosolic materials, TNTs can allow the transfer of membrane components such as cell surface receptors and membrane-anchored proteins. In their first description of TNTs, Rustom *et al*. showed the transfer of membrane-bound (farnesylated) Ras to a connected PC12 neuronal cell^[41]^. Cell surface MHC-I can be present on nanotubes and be transferred between immune cells^[75,90]^. Additionally, surface-expressed CD59 and CD81 have also been reported to be exchanged bidirectionally between Jurkat T-cells^[80]^.

Labeling of intracellular vesicles with lipophilic dyes and immunostaining with vesicle/organelle-specific markers have proven to be reliable approaches in assessing vesicular transfer between donor and acceptor cells via TNTs. Endosomes, lysosomes, mitochondria, Golgi, and endoplasmic reticulum have been reported to utilize TNTs for transfer between cells (reviewed in^[91]^). However, the intercellular exchange of mitochondria has been of particular interest because of their potential involvement in regulating the metabolism of acceptor cells and alleviating the health of diseased cells^[83,92,93]^. Mitochondrial dysfunction in dopaminergic neurons has been shown to be attenuated by transfer of functional mitochondria from astrocytes^[94]^. In the presence of pathogenic load of *α*-Syn in astrocytes, healthy mitochondria are transferred via TNTs from neighboring astrocytes as a way of restoring homeostasis^[95]^. Recent findings also suggest that mitochondria move in TNTs from microglia to neuronal cells in co-culture, preferably to α-Syn loaded cells, compared to healthy cells^[96]^. Besides mitochondria, lysosomes actively move between cells via TNTs^[41,97]^. Such transfer provides a route for the movement of *α*-Syn aggregates that hitchhike functionally compromised lysosomes to spread and propagate *α*-Syn pathology^[70]^.

### Pathogens

Several pathogens such as bacteria and viruses utilize TNTs as a route of propagation. In the earliest description, *Mycobacterium bovis* bacillus Calmette-Guérin (BCG) has been reported to move between human monocyte-derived macrophages^[40]^. Human Immunodeficiency virus (HIV) facilitates its spreading by inducing TNT formation, eventually associating with endosomes and MVBs for transfer between macrophages^[98]^. Recently, SARS-CoV-2 has been shown to utilize TNTs to spread between permissive epithelial and non-permissive neuronal cells^[46]^, implicating a potential role of these structures in the manifestation of neurological symptoms upon viral infection.

## TUNNELING NANOTUBES VERSUS EVS IN THE SPREAD OF NEURODEGENERATIVE PATHOLOGIES

With accumulation of protein aggregates and concomitant compromise of quality control pathways in NDs^[99]^, burdened cells remain far from homeostasis. The inability of post-mitotic cells like neurons to dilute out these protein aggregates eventually leads to their degeneration, besides the non-cell-autonomous effects of glial cells in neurotoxicity^[100]^. With the progression of the pathologies (Braak’s stages)^[99]^, degeneration spreads from the epicenter of initial aggregate seeding to different regions of the brain. A major mechanism of such spreading is via secretory pathways, with the release of protein aggregates in extracellular vesicles that are eventually internalized by other cells. The close association of protein aggregates with exosomes has been reported for several NDs (reviewed in^[100]^). Both soluble and aggregated forms of Prion (PrP^C^ and PrP^Sc^, respectively) associate with exosomes, with PrP^Sc^ causing aggregation in the acceptor cell^[101]^. In Alzheimer’s disease (AD), both tau and amyloid precursor protein (APP), associated metabolites and secretase enzymes have been shown to be present in exosomes^[102,103]^. Parkinson’s disease (PD) causing *α*-Syn can be packaged within exosomes and eventually secreted^[104]^. Such exosomes are internalized by neighboring cells, preferably over isolated oligomers not associated with exosomes^[105]^. In Amyotrophic Lateral Sclerosis (ALS), both WT and mutant Superoxide dismutase (SOD1) associate with exosomes and are released by both neurons and astrocytes *in vitro* and *in vivo*^[106-108]^. Exosomes have also been reported to transport transcripts of mutant *huntingtin* (*mHtt*), besides the protein itself, in the case of HD^[109,110]^. Although these studies confirm the propagative roles of exosomes in different NDs, it is important to note that purification and/or concentration of secreted vesicles does not represent the true extent of secretion-mediated transfer. Additionally, secretion-based mechanisms are not the exclusive routes for the spread of such pathologies. In the subsequent sections, we discuss a parallel mechanism in place for aggregate transfer between cells.

**Figure 3:**
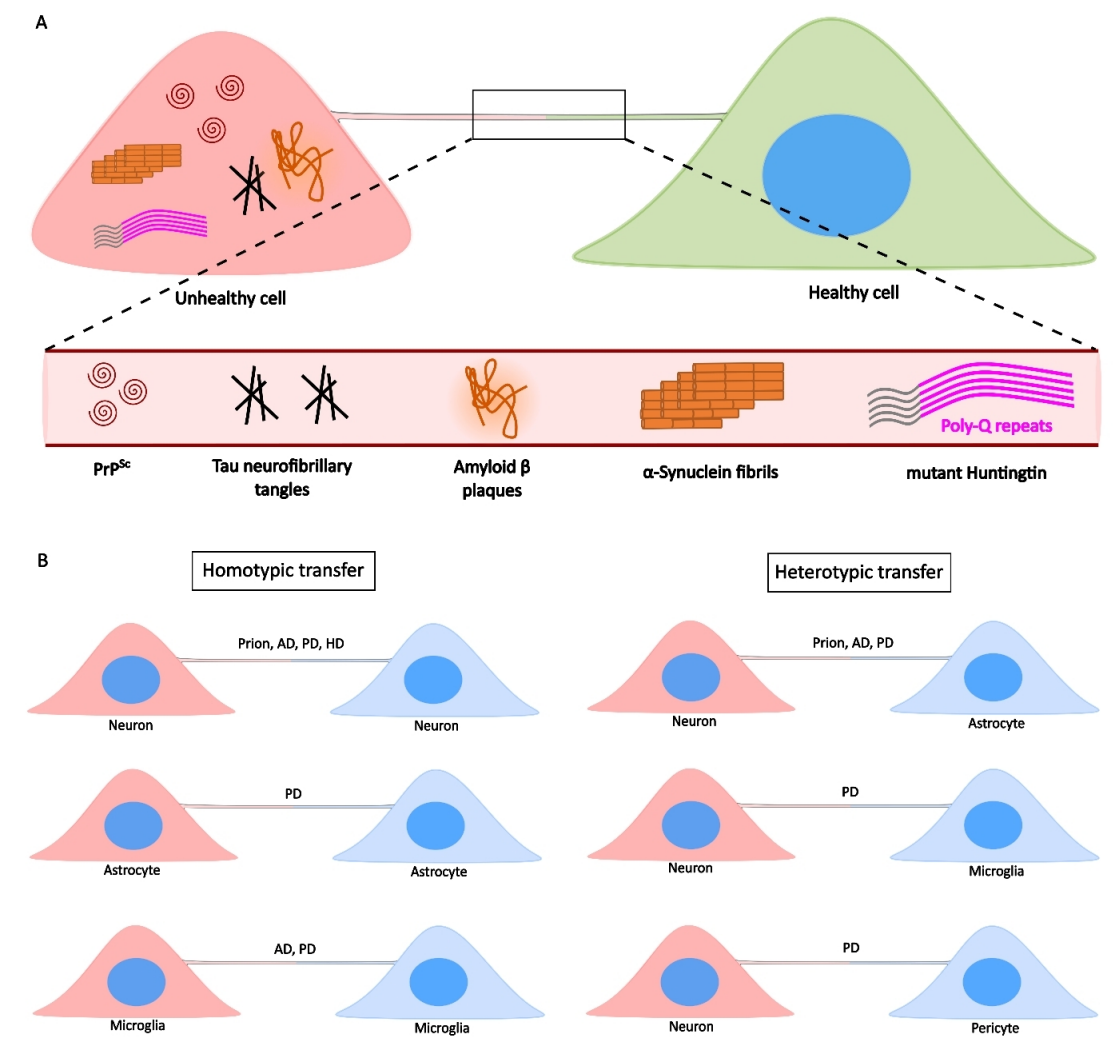
TNTs facilitate the transfer of NDs-causing protein aggregates. A: Unhealthy cells containing protein aggregates can form TNTs with a naïve, healthy cell, eventually spreading aggregates such as PrP^Sc^ (Prion’s disease), Tau and Amyloid-*β* (AD), *α*-Syn fibrils (PD), and mHtt (HD). B: Such transfers can happen between the same type of cells via homotypic TNTs (left panels), or between different cell types via heterotypic TNTs (right panels).

A major shift in the paradigm of neurodegenerative pathology spreading happened when TNTs were shown to transfer PrP^Sc^ between neuronal cells^[97]^. This study paved the way for several subsequent reports on different types of protein aggregates utilizing TNTs as a route for spreading to neighboring cells, both neuronal and non-neuronal [Figure 3A-B, and Table 1]. Not only are different aggregates transferred between cells, but there also happens an increase in the extent of TNT-mediated intercellular connectivity in the presence of neurotoxic aggregates. As such, an existent dogma in the field suggests that protein aggregates increase TNTs as a way of increasing transmissivity between cells. We and others have proposed that this phenomenon might be linked to an increase in ROS species in cells burdened with toxic aggregates^[48]^. However, more studies will be needed to understand the precise mechanism(s) and molecular pathways leading to TNT increase in these conditions.

**Table 1:** Involvement of TNTs in NDs

**ND pathology**	**TNT involvement**	**Material transferred**	**Cell types involved**	**Reference**
Prions Disease	Yes (pathology spreading)	PrP^C^, PrP^Sc^	CAD neuronal cells Dendritic cells-primary neurons Neurons-astrocytes	[97] [97,112] [114]
AD	Yes (pathology spreading)	A*β* Tau	Astrocytes SH-SY5Y neuronal cells Microglia HeLa CAD neuronal cells Primary neurons Neurons-astrocytes (organotypic cultures) Microglia	[115] [116] [119] [117] [71,117] [71,118] [71] [119]
PD	Yes (pathology spreading, and aggregate clearance)	*α*-Syn	CAD neuronal cells Primary neurons Human NPCs Murine astrocytes Murine neurons-astrocytes Human astrocytes SH-SY5Y neuronal cells-primary human brain pericytes Murine microglia Human PBMC-derived microglia-like cells Microglia (*in vivo*) SH-SY5Y neuronal cells-HMC3 microglial cells	[120] [120] [121] [69] [69] [95] [122] [119] [119] [119] [96]
HD	Yes (pathology spreading)	Rhes, mHtt	CAD neuronal cells Primary cerebellar granule cells Mouse normal striatal neuronal cells Primary striatal neurons Striatal medium spiny neurons (*in vivo*)	[123] [123] [124] [124] [125]

### Prion’s Diseases

Prion proteins can cause Creutzfeldt-Jakob disease (CJD), Gerstmann-Sträussler-Scheinker disease (GSS), and fatal familial insomnia (FFI) in humans. Over the years, prions have gained considerable attention because of their ability to be transmitted from animals to humans, as seen in the case of bovine spongiform encephalopathy (BSE)^[111]^. The aggregate-prone form of Prion, PrP^Sc^, has been reported to transfer via TNTs not only between neuronal cells, but also between bone marrow-derived dendritic cells and primary neurons^[97,112]^. PrP^Sc^ increases the formation of TNTs between neighboring cells, possibly by causing ER stress and differential distribution of membrane cholesterol, and eventually makes its way to a different cell by “hijacking” the endo-lysosomal vesicles^[113]^. Besides neuronal cells, astrocytes can also form TNTs with neurons in order to transfer vesicles containing PrP^Sc[114]^, thereby contributing to a global spread of pathogenicity between neuronal and glial cells.

### Alzheimer’s Disease

A major hallmark of AD pathology is the presence of both extracellular amyloid beta (A*β*) plaques and intracellular Tau neurofibrillary tangles. As such, a critical question that arises is whether both A*β* and Tau are transferred inter-cellularly via TNTs. Wang *et al*. were the first to report TNT-mediated transfer of A*β* between neurons and astrocytes *in vitro*. Although the transfer of A*β* failed to increase the number of TNTs, it induced cytotoxicity in the acceptor cells. Additionally, the stressed cells were the initiators of TNT formation with a healthy cell in a p53-dependent manner^[115]^. A recent report suggests that oligomeric A*β* (1-42) induce TNT formation between undifferentiated and partially-differentiated SH-SY5Y neuronal cells that is dependent on the Actin regulatory kinase PAK1, which allows for transfer of oligomers between connected cells^[116]^. In addition to A*β*, Tau propagation via TNTs has also been reported. Tau fibrils increased the number of TNTs between HeLa cells, CAD neuronal cells, and primary neurons, allowing for intercellular transfer of these aggregates^[117,118]^. Recently, Tau propagation via TNTs followed by aggregate seeding in acceptor cells has been reported between primary neurons, as well as between neurons and astrocytes in organotypic culture system^[71]^. Altogether, these reports suggest the crucial role of TNTs in the spread of AD-causing protein aggregates. Interestingly, microglia share both A*β* and Tau fibrils amongst themselves, albeit to a much lesser extent than *α*-Syn aggregates^[119]^.

### Parkinson’s Disease

PD pathology manifestation is majorly associated with aggregation of *α*-Syn in neurons, leading to cellular death. Propagation of aggregates has been observed via both secreted and contact-mediated mechanisms. *α*-Syn fibrils were first reported to utilize TNTs as a route for spreading between CAD neuronal cells and primary neurons^[120]^. *α*-Syn fibrils have also been shown to be transferred between human neural progenitor cells (hNPCs) via TNTs^[121]^. A mechanism of aggregate transfer involves de-functionalization of lysosomes by fibrils, which are then transferred together via TNTs^[70]^. Eventually, several other studies have reported the involvement of TNTs in mediating neuron-glia and glia-glia transfer of such aggregates. *α*-Syn fibrils can be transferred between murine astrocytes, as well as from neurons to astrocytes, wherein the fibrils are eventually degraded^[69]^. On the other hand, experiments with human astrocytes have revealed that aggregates transferred between them are incapable of being degraded, under which circumstances TNTs could contribute to spreading of the pathology^[95]^. Additionally, TNTs connecting SH-SY5Y neuronal cells with primary human brain pericytes facilitate the movement of *α*-Syn between them^[122]^. Murine primary microglia and human peripheral blood mononuclear cells-derived microglia form extensive intercellular networks of TNTs that contribute to the movement of *α*-Syn fibrils between cells^[119]^. Our recent work also highlights the movement of such fibrils preferentially from neuronal to microglial cells via TNTs. The extent of TNT-mediated fibril movement from neuronal to microglial cells was significantly higher than in the other direction, implicating a significant role of TNTs in mediating neuron-glia communication^[96]^. These results altogether provide a significant resource for understanding PD pathology spread between different cell types of the brain. However, a comprehensive understanding of the (patho)physiological significance of such differences in transfer and its contribution to PD spread in human brains is currently lacking.

### Huntington’s disease

HD manifestation occurs due to extensive “CAG” repeats in exon 1 of the *Huntingtin* gene, which generates mutant protein. Although not a lot of attention has been paid to the extent of TNT-mediated transfer of mHtt between cells, there exist several lines of evidence, *in vitro* and *in vivo*, that confirm the involvement of TNTs in the spread of pathology. mHtt movement between CAD neuronal cells and primary cerebellar granule neurons has been reported to occur via TNTs^[123]^. Subsequent reports from the group of Subramaniam have shown the involvement of Rhes protein in the regulation of TNT formation and transfer of mHtt between striatal neuronal cell lines and primary neurons, which the authors referred to as “Rhes tunnels”^[124]^. *In vivo*, transfer of mHtt between medium spiny neurons of striatum, as well as from striatum to cortex, is reported to be dependent on Rhes-mediated connections between cells^[125]^. With the understanding of Rhes-regulated mHtt transfer via TNTs, it would be interesting to assess the roles of glial cells in mHtt pathology spread.

## CONCLUSION AND FUTURE PERSPECTIVES

The discovery and consecutive studies on TNTs in the past decades have enlarged the picture of intercellular communication mechanisms. However, in order to understand the challenges to overcome in the future, it is important to recognize the limitations of the field. First, TNTs are fragile structures and do not survive most fixation protocols. A recently published paper used microfluidics and AFM indentation to display the elastic properties of TNTs in human embryonic kidney cells, enabling them to resist bending^[126]^. They revealed that TNTs formed between cells separating faster than 0.5 µm/min are highly unstable. The frailty of these structures might have initially represented an important limitation for the field, but nowadays, protocols for fixation, identification and characterization of TNTs have been well documented^[127]^. Another issue that has been extensively addressed is the lack of specific markers to distinguish TNTs from other TNT-like structures. Their ability to transfer vesicles and organelles between cells is unique. However, even though live imaging of such transfer is critical to demonstrate their functionalities, the low frequency of these events does not allow for robust quantification. Currently, thorough studies on TNTs rely on the combination of several parameters to distinguish them from filopodia in fixed and live samples for quantification: they should hover over the substrate, have a length above 10 μm, contain Actin and have a diameter below 1 μm. The combination of these parameters serves the purpose of decreasing the number of false positives when studying TNTs, as confusion between filopodia and TNTs is the main bias to avoid. A major drawback of such a method is the increase in false negatives. This analysis allows the identification of a specific subtype of long, non-adherent TNTs, yet does not consider structures too close to the substrate or smaller than 10 μm^[55-68]^. Finally, the field of TNTs faces the same challenge as studies on EVs, or even filopodia. The difficulty of purifying these structures and the complexity of the phenomenon studied requires us to categorize them using distinct terminology. Therefore, terms such as “exosome”, “ectosome”, “migrasomes”, “filopodia”, or “TNT”, are likely to encompass a multitude of structures with important structural and functional differences, as suggested by numerous studies in the field^[13,128,129]^. In other words, semantics could provide a biased perception of the actual processes undergoing in living cells. The molecular similarities of the formation mechanisms of TNTs, filopodia and EVs, along with the diversity of protein and lipid compositions within each of these, call for caution when using these nomenclatures because of likely molecular overlap, yet considerable (yet unknown) differences in function/regulation.

Overall, the concomitant role of EVs and TNTs in inflammation and immune response, along with the overlap we can observe in the molecular actors involved in their formation (IRSp53, CD9/CD81), suggests the studies of EVs could help us improve our understanding of TNTs and vice versa, by the identification of common actors and regulators. Determining where their molecular pathways cross and divide represents a key objective for both the fields of TNTs and EVs. 

As discussed above, TNTs are capable of transferring a myriad of intracellular materials from one cell to another. In physiological contexts, while transfer of Ca^2+^ can allow electrical coupling and subsequent development of immature cells, movement of “death signals” can bring about a global senescent response by the network of connected cells. Similarly, movement of mitochondria can help rescue an apoptotic cell, while movement of damaged lysosomes containing protein aggregates can spread neurodegenerative pathologies. Quite recently, mitochondrial movement has been reported to occur from mesenchymal stem cells to neurons as a potential protective mechanism in place^[130]^. Although we focused on the roles of TNTs in pathological spread of toxic protein aggregates, the influence of secretion in disease spread is significant. Several protein aggregates are released by cells that can be taken up by neighboring cells, or accumulate extracellularly. It is highly plausible that there exists a concerted mechanism (yet unknown) of secretion- and TNT-based intercellular communication that takes place in symbiosis to cause ND spread. Our limited understanding of TNTs *in vivo* also poses a challenge in understanding not only ND progression across different Braak stages^[101]^, but also in testing the potential interplay of secreted vesicles and TNTs in disease progression. Future studies would require assessing the presence of TNTs in healthy and diseased brains, with stringent emphasis on characterization of the structures as TNTs or TNT-like.

As such, TNTs have been rightly referred to as a “double-edged sword” that can be both beneficial and detrimental, depending on the context of formation. This raises important questions about to what extent is the movement of context-specific molecules between healthy and unhealthy cells actively regulated, and what are the molecular players involved in “sensing” which material(s) to transfer.

## DECLARATIONS

### Copyright

© The Author(s) 2023.
